# Associations of prodynorphin sequence variation with alcohol dependence and related traits are phenotype-specific and sex-dependent

**DOI:** 10.1038/srep15670

**Published:** 2015-10-27

**Authors:** Stacey J. Winham, Ulrich W. Preuss, Jennifer R. Geske, Peter Zill, John A. Heit, Georgy Bakalkin, Joanna M. Biernacka, Victor M. Karpyak

**Affiliations:** 1 Health Sciences Research, Mayo Clinic, Rochester, MN; 2 Department of Psychiatry, Psychotherapy and Psychosomatics, Martin-Luther-University of Halle-Wittenberg, Halle/Saale, Germany; 3 LMU Munich, Department of Psychiatry, Section Psychiatric Genetics and Neurochemistry, Munich, Germany; 4 Psychiatry and Psychology, Mayo Clinic, Rochester, MN; 5 Division of Cardiovascular Diseases, Mayo Clinic, Rochester, MN; 6 Division of Biological Research on Drug Dependence, Department of Pharmaceutical Biosciences, Uppsala University, Uppsala, Sweden

## Abstract

We previously demonstrated that prodynorphin (*PDYN*) haplotypes and single nucleotide polymorphism (SNP) rs2281285 are associated with alcohol dependence and the propensity to drink in negative emotional states, and recent studies suggest that *PDYN* gene effects on substance dependence risk may be sex-related. We examined sex-dependent associations of *PDYN* variation with alcohol dependence and related phenotypes, including negative craving, time until relapse after treatment and the length of sobriety episodes before seeking treatment, in discovery and validation cohorts of European ancestry. We found a significant haplotype-by-sex interaction (p  =  0.03), suggesting association with alcohol dependence in males (p = 1E-4) but not females. The rs2281285 G allele increased risk for alcohol dependence in males in the discovery cohort (OR = 1.49, p = 0.002), with a similar trend in the validation cohort (OR = 1.35, p = 0.086). However, rs2281285 showed a trend towards association with increased negative craving in females in both the discovery (beta = 10.16, p = 0.045) and validation samples (OR = 7.11, p = 0.066). In the discovery cohort, rs2281285 was associated with time until relapse after treatment in females (HR = 1.72, p = 0.037); in the validation cohort, it was associated with increased length of sobriety episodes before treatment in males (beta = 13.49, p = 0.001). Our findings suggest that sex-dependent effects of *PDYN* variants in alcohol dependence are phenotype-specific.

Converging evidence from experimental studies and postmortem human brain research indicate that the dynorphin/kappa-opioid receptor system plays an important role in alcohol and drug dependence^1^,^2^,^3^,^4^. Genetic findings indicate that sequence variations in *PDYN* and *OPRK1* genes—which encode dynorphins and the kappa-opioid receptor, respectively—are associated with risk for alcohol dependence^5^,^6^, as well as opioid and cocaine dependence^7^,^8^. We recently demonstrated that *PDYN* haplotypes are associated with increased risk for alcohol dependence and propensity to use alcohol in order to relieve negative emotions (negative craving)^9^. We also found that the minor G alleles of rs2281285 in the second intron and rs6132153 downstream of *PDYN* exhibited trends towards association with both phenotypes^9^. In a separate study sample, we demonstrated that the rs2281285 G allele is also associated with drinking in order to avoid somatic or emotional discomfort associated with alcohol withdrawal^10^–another phenotype associated with negative craving^11^.

Negative (or ‘relief ’) craving is defined as the desire for drinking (craving) in the context of tension or negative emotions, in contrast to positive (or ‘reward’) craving defined as the desire for the rewarding properties of alcohol, or obsessive thoughts about drinking (obsessive or ‘temptation’ craving), conceptualized in a three-pathway psychobiological model^11^. According to this model, negative/relief craving reflects dysregulation in the glutamate/GABA balance in favor of excitation excess, while positive/reward craving reflects dysregulation in dopamine/opioid neurotransmission, and obsessive/temptation craving is attributed to dysregulation in serotonin neurotransmission^11^. The evolving understanding of the complexity of opioid neurotransmission and the role of dynorphin/kappa-opioid receptor signaling in negative emotions and addiction^12^,^13^ and our findings associating *PDYN* variation with negative craving and alcohol withdrawal^9^,^10^, indicate that biological underpinnings of each contextual subtype of craving requires further investigation.

Subtypes and patterns of manifestation of alcohol and substance dependence are also known to be sex-dependent. For example, research findings indicate higher rates of alcohol dependence in males^14^; however, females report tendency to drink in response to unpleasant emotions more often than males, and these associations are mediated by depression severity^15^. Our data indicate a stronger tendency towards heavy drinking in unpleasant emotional situations in female compared to male alcoholics^16^. Recently, it has been recognized that sexual dimorphism in disease may arise through evolutionary mechanisms (such as sexual selection and sexual antagonism)^17^, resulting in sex-dependent genetic architecture^18^,^19^; in fact, heritability estimates for alcohol dependence are higher in males than females, indicating that genetic risk may be sex-dependent^14^.

Importantly, experimental studies indicate sex-related differences in sensitivity to depressive-like effects of the kappa opioid receptor agonist^20^. Evidence also indicates that sequence variants within the *PDYN* gene modulate its expression and that effects on transcription differ by brain region, sex, and cell type^21^. Moreover, experimental data indicates that *PDYN* expression in brain and ovarian tissue is modulated by dihydrotestosterone and gonadal steroid hormones^22^,^23^, and that *PDYN* mRNA levels, as well as cocaine-induced behaviors, may be modulated by estrogen and progesterone^24^. Therefore, it is possible that effects of *PDYN* variants on development of alcoholism and related phenotypes may also differ in males and females, resulting in SNP by sex interactions and sex-dependent genetic effects.

In fact, a sex-specific association between the C allele of downstream *PDYN* variant rs1022563 and risk of heroin dependence has been reported^25^,^26^. According to the 1000 Genomes Project Phase 1 CEU data, in populations of European ancestry, rs1022563 is in high linkage disequilibrium (LD, R^2^ = 0.81) with rs2281285, and is also in perfect LD (R^2^ = 1) with rs6132153--SNPs which we found to be associated with alcohol dependence and negative craving in European American subjects^9^. Therefore, it is possible that these *PDYN* variants exhibit related sex-dependent effects across substance-dependence and related traits.

To test these hypotheses, we re-analyzed our previously reported data by examining SNP-by-sex interaction effects on alcohol dependence and negative craving. We also conducted additional analyses focused on related phenotypes, including time to relapse after treatment and the length of sobriety episodes before seeking treatment.

## Methods

### Discovery Sample

This work was approved by the Institutional Review Board of the Mayo Clinic, Rochester, Minnesota and was conducted according to the Code of Ethics of the World Medical Association (Declaration of Helsinki). All participants signed informed consent and gave permission for the use of their information in future research of alcohol dependence and related phenotypes. Characteristics of the samples and genotyping procedures have been described previously^9^,^27^. In brief, the discovery cohort consisted of 816 alcohol-dependent cases and 1248 controls recruited from alcohol dependent subjects and non-alcohol dependent controls treated in programs affiliated with Mayo Clinic, Rochester, Minnesota^9^. The Illumina GoldenGate SNP assay was used for genotyping in alcohol dependent subjects (cases), and included 13 *PDYN* variants (chosen to match the 13 *PDYN* variants previously genotyped in controls) and 43 ancestry informative markers^28^ used to verify self-reported race. The genotyping data for control subjects was retrieved from a previously published genome wide association study using the Illumina 660 genome-wide SNP array^29^. While potential control subjects with a history of alcohol dependence were excluded, controls were not required to have a history of exposure to alcohol. Controls were not matched to cases on age and sex, and were older and included more females^9^.

Because *PDYN* has undergone positive selection, minor allele frequencies for *PDYN* variants differ substantially across human subpopulations^30^, potentially increasing the possibility for spurious associations due to population stratification. To avoid this risk, analyses were restricted to subjects with European ancestry, which were verified with the use of ancestry informative markers. Both discovery and validation cases were recruited from subjects actively enrolled in alcohol treatment programs (see Supplementary Materials for a description of the treatment programs). Clinical and demographic characteristics of the alcohol dependent subjects included in the discovery and validation cohorts are described below and summarized in Table 1.

In the discovery cohort, presence of alcohol dependence was determined in a clinical assessment by board certified addiction psychiatrists and defined based on DSM-IV-TR criteria^31^. Negative craving was measured by the negative sub-scale of the Inventory of Drug Taking Situations (IDTS)^32^. Relapse after treatment was defined as a return to any alcohol use following completion of treatment. Time until relapse was measured in the 12 month period following standard treatment. Relapse/sobriety data are collected as a part of clinical follow up at 3, 6, 9 and 12 month intervals after completing standard treatment.

### Statistical Analysis

Our previous study indicated that the effects of *PDYN* on alcohol dependence were driven by rs2281285^9^, so this variant was the primary focus of analysis. In the discovery cohort, we performed single SNP association tests for the previously reported rs2281285 variant. We assumed additive genetic effects, coding SNP genotypes as the number of copies of the minor allele (0–1–2). We evaluated sex-related effects on risk for alcohol dependence of rs2281285 by examining a SNP-by-sex interaction under a logistic regression model, adjusted for age and sex. Similarly, we examined the SNP-by-sex interaction of rs2281285 on negative craving using linear regression (adjusted for age and sex) in a subsample of N = 196 cases with available IDTS data. In addition to phenotypes investigated in our previous publications, we also examined the SNP-by-sex effects of rs2281285 on time until relapse (in months) in a subset of N = 202 case subjects with 12 months of follow-up data after treatment using Cox proportional hazards regression (adjusted for age and sex). Along with tests of SNP-by-sex interaction, analyses were also conducted adjusted for age and sex (without interaction), as well as adjusted for age and stratified by sex. Because the studied phenotypes of interest are highly correlated (and therefore cannot be considered independent tests), no standard multiple testing correction was appropriate; reported p-values are uncorrected. Instead, we used validation in the independent sample (see below) as a way to determine robustness of association findings. All statistical analysis was performed in R Statistical Software, version 2.14.0 (http://www.r-project.org).

### Sensitivity Analyses

As sensitivity analyses, the single SNP analyses of rs2281285 were repeated with the variant rs6132153, which is strongly linked (R^2^ = 0.82 in controls); this variant is in complete LD with another variant previously associated with opioid dependence^25^. In the discovery cohort, we also examined previously identified haplotypes^9^ as sensitivity analyses, because haplotype analyses may be more powerful if an un-genotyped marker in linkage disequilibrium (LD) with rs2281285 is actually causal. Association tests of haplotype-by-sex interaction were adjusted for age and sex. Additionally, haplotype association tests were also performed adjusted for age and stratified by sex. We examined a previously identified *PDYN* gene-spanning haplotype (rs6045868-rs2235751-rs2281285) associated with alcohol dependence^9^ using logistic regression. Similarly, we examined the haplotype-by-sex interaction of a previously identified *PDYN* haplotype (rs6045784-rs910080-rs2235751-rs2281285)^9^ on negative craving using linear regression, and time until relapse using Cox proportional hazards regression. Haplotype association tests were performed using the score statistic proposed by Schaid *et al.*^33^, and empirically derived p-values are reported for the global test (4 degrees of freedom for the three SNP haplotype, and 5 degrees of freedom for the four SNP haplotype) and for each individual haplotype. Haplotypes were estimated and all haplotype analyses were performed using the R package ‘haplo.stats’ (http://cran.r-project.org/web/packages/haplo.stats/). As the haplotype and analyses and analyses of rs6132153 are highly correlated with the analyses of rs2281285, no additional multiple testing correction was applied.

### Validation Sample

The validation cohort has been described in detail in a previous publication^27^. In brief, patients 18 years and older were consecutively recruited from an inpatient addiction treatment unit of the Department of Psychiatry, Ludwig-Maximilians University, Munich, Germany (see Supplementary Material). All study subjects met DSM IV criteria for alcohol dependence and were assessed with the Semi-Structured Interview for the Assessment on the Genetics of Alcoholism (SSAGA)^34^,^35^. All participants provided written informed consent approved by the ethical committee of the Ludwig-Maximilians University. Collected DNA samples were genotyped for rs2281285 only using a TaqMan® SNP Genotyping Assay in 467 alcohol dependent subjects and 431 controls, as described in our previous publication^9^.

In our previous publications, we examined the effects of rs2281285 on alcohol dependence and negative craving using this sample^9^,^10^, although sex-specific effects were not explored. In the present study, we used the validation cohort to examine the sex-dependent effects of rs2281285 on alcohol dependence using logistic regression, adjusted for age. The same measures of negative craving and time to return to drinking used in the discovery sample were not available in the validation sample, limiting the ability for direct replication; however, available measures assessing similar constructs were utilized to validate the findings of the discovery cohort. Specifically, because the IDTS assessment was not performed in this cohort, negative craving was instead assessed with a binary SSAGA item asking if the patient ever ingested alcohol to avoid unwanted emotional or somatic discomfort^10^; sex-specific effects of rs2281285 were assessed with logistic regression, adjusted for age. Furthermore, data on time until relapse after treatment for alcohol dependence was not available in this sample. Therefore, a related variable of length of sobriety before seeking treatment was utilized to assess association with rs2281285 using age-adjusted linear regression. The choice of this variable was based on an assumption that vulnerability to negative craving associated with *PDYN* variation may cause earlier return to drinking at any time, not just after treatment.

## Results

### Descriptive Statistics

Description of demographic characteristics of the study cohorts and investigated alcoholism-related phenotypes are presented in Table 1. Both discovery and validation cohorts were recruited from subjects enrolled in treatment programs and included more males then females. However, the validation cohort was younger (p < 0.0001), had heavier alcohol consumption (p < 0.0001), and a higher percentage of males (p = 0.018); analyses were adjusted for age and sex in both sample sets, but differences in data collection methods prohibited adjustment for consumption in the discovery sample. The discovery cohort had a larger sample size of cases (N = 816) and controls (N = 1248) to test associations between *PDYN* variants and alcohol dependence compared to the validation cohort (N = 467 and 431, respectively). However, smaller numbers of participants in the discovery cohort were assessed with the negative subscale of IDTS (N = 196) and had available post-treatment relapse data (N = 202). In the validation cohort, response to the SSAGA question about drinking to avoid emotional/physical symptoms of withdrawal (yes/no) and data about maximum length of sobriety before treatment were available for most subjects (N = 417 and 409, respectively).

### Alcohol Dependence

The SNP-by-sex interaction effect of rs2281285 on alcohol dependence did not reach a statistically significant level but showed a trend towards association in the discovery cohort (p = 0.099), indicating that the effect of rs2281285 on risk for alcohol dependence may depend on sex. Subsequent stratified analyses examining the SNP effect separately in males and females yielded consistent results in the discovery and validation cohorts. As shown in Tables 2 and 3, we observed a stronger association between risk for alcohol dependence and the rs2281285 G allele in males in both the discovery (OR = 1.49, p = 0.002) and the validation cohort (OR = 1.35, p = 0.08), but no significant association in females.

### Negative Craving

Conversely, sex-stratified analyses of rs2281285 on negative craving showed an association in females but not males (Tables 2 and 3). In females, the G allele of rs2281285 was associated with increased negative craving in the discovery sample (beta = 10.16, p = 0.05; Table 2), and trended towards association with avoidance of unwanted emotional and somatic discomfort in the validation sample (OR = 7.11, p = 0.07; Table 3).

### Return to Drinking

Similarly, the rs2281285 G allele was associated with increased risk of post-treatment relapse (OR = 1.42, p = 0.048) in the discovery cohort (Table 2), where the association is driven by females (HR = 1.72, p = 0.037) but not males. In the validation cohort (Table 3), the minor rs2281285 G allele was associated with an increased length of sobriety of 10 months on average (p = 0.02), but the effect is driven by males (beta = 13.5, p = 0.001).

### Sensitivity Analyses

Single SNP results for rs6132153 were similar to those for rs2281285 for all phenotypes in the discovery cohort (Supplemental Table 1). Additionally, we found a statistically significant haplotype-by-sex interaction effect on alcohol dependence in the discovery cohort (p = 0.03). In a sex-stratified analysis (Supplemental Table 2), the rs6045868-rs2235751-rs2281285 haplotype was associated with alcohol dependence in males (p = 1E-4) but not females. There was no significant *PDYN* gene-spanning haplotype-by-sex interaction effect on negative craving or time until relapse after treatment.

## Discussion

Results presented here demonstrate that our previously reported findings of association between *PDYN* SNPs and haplotypes and alcohol dependence may be sex-related, and importantly, the sex-dependent effects are also trait (phenotype)-specific. A significant haplotype-by-sex interaction was observed for alcohol dependence, and stratified analyses indicated that the effect of the *PDYN*-spanning haplotype was present in males only. However, the SNP-by-sex interaction for rs2281285 was only marginally significant, indicating that perhaps rs2281285 is not the causal variant, but rather is in LD with a causal variant (or multiple variants) that is also tagged by the *PDYN*-spanning haplotype. Similarly to the results of the haplotype analysis, the effect of the minor G allele of rs2281285 was stronger in males in both discovery and validation cohort analyses, and the male-specific effect observed in the discovery cohort was statistically significant. This effect was consistent in the direction and effect size in the validation cohort, where, despite smaller sample size, we also observed a trend for association of the rs2281285 G allele with increased risk for alcohol dependence in male but not in female alcoholics. Moreover, in sex-specific analyses of the effect of rs2281285 on negative craving, we observed a female-specific trend for association between the rs2281285 minor G allele and increased negative craving in both the discovery and validation cohorts. Similarly, the minor rs2281285 G allele was associated with increased risk of relapse in female subjects in the discovery cohort, while in the validation cohort this same allele was associated with increased length of sobriety before treatment in male alcoholics.

Notably, the genetic effects detected here have not been previously implicated in genome-wide association studies of alcohol dependence^36^,^37^,^38^,^39^,^40^. Many of these studies did not investigate SNP-sex interactions or perform sex-stratified analyses; and for prior studies that examined sex strata (or included males only), sample sizes were small. Our results demonstrating sex-specific genetic effects on alcohol related traits suggest that effects for some SNPs may be larger in one sex; by analyzing males and females together, the effects would be reduced and may not be detected. In light of evolutionary mechanisms underlying sex differences in disease risk^18^, it is likely that interactions between genetic variants and sex exist, and ignoring such interactions in analyses can prevent detection of certain genetic effects^41^.

The findings of this study are intriguing, as the minor alleles of the *PDYN* SNPs rs2281285 and rs6132153 seem to have different effects on risk for alcohol dependence, negative craving and the length of sobriety before and after treatment for males and females. Specifically, the minor G alleles of these genetic variants convey increased risk for alcohol dependence in males, while increasing vulnerability to negative craving and post-treatment relapse in alcohol dependent females. One possible explanation for these findings is that the above mentioned genetic variants are associated with an intermediate phenotype, which is more common in alcoholic males compared to non-alcoholic males, and even more common in alcoholic females. Increased stress vulnerability as well as comorbidity of alcohol dependence with depression and/or anxiety are among potential candidates for such an intermediate phenotype(s). In fact, clinical and epidemiological studies indicate that comorbid depression or anxiety are more common in alcoholics compared to the general population, and are also more common in alcoholic females than males^42^,^43^,^44^, while alcohol consumption in the context of negative emotional states (negative craving) is also known to be associated with female sex^15^,^16^.

Importantly, the presence of comorbid mood and anxiety disorders in alcohol dependent subjects is reported among empirically described subtypes of alcohol dependence, which are also characterized by persistence of symptoms, treatment seeking and worse mental health status^45^,^46^. Moreover, evidence indicates that comorbidity of alcohol dependence with mood and anxiety disorders appears to be attributable to factors shared among these disorders^47^. Although speculative at this point, it is possible that the sex-related effects of the *PDYN* variants reported here are narrowing in on the genetic underpinnings of these particular subtypes of alcohol dependence. Similarly, other traits relevant to alcohol dependence that may differ between males and females should also be considered, as additional factors may be involved in higher-order (perhaps endophenotypic) interactions with SNP and sex effects on alcohol dependence, and may define important sex-associated subtypes. For example, negative craving is associated with length of time abstinent from drinking^48^, and our data suggests that both sex and *PDYN* variants may modify these relationships. These hypotheses regarding the role of comorbidities, and how they may have impacted the observed associations, should be investigated in the future studies.

Several limitations to the current study need to be acknowledged. First, sample size limited power to detect gene-sex interactions and sex-specific effects. Fewer female subjects in both the discovery and validation cohorts resulted in lower power for female-specific compared to male-specific analyses, and because the discovery and validation samples were selected with alcohol dependence as the primary phenotype of interest, sample sizes and power to detect associations with the secondary case-only phenotypes were reduced. Furthermore, the validation sample was smaller than the discovery sample with respect to the primary phenotype, which also limited power.

Second, the controls in the discovery cohort were previously ascertained from a sample of control subjects representing the general population. This has become a common strategy in study of genetic epidemiology^49^. However, because of this, cases and controls were genotyped separately (although subjects genotyped in both sets were 100% concordant) and controls were not matched to cases on either age or sex. To mitigate this difference, analyses were adjusted for age and sex to eliminate potential biases due to these factors. Although control subjects with a history of alcohol dependence were excluded, controls were not required to have a history of exposure to alcohol. We do not expect this approach to induce an ascertainment bias, but perhaps further reduce power, because the control population may include subjects with high genetic risk who may have developed alcohol dependence after exposure^50^.

Third, because the phenotypes investigated here are highly correlated, the analyses we conducted were highly dependent. Therefore, multiple testing correction methods (e.g. Bonferroni correction or false discovery rate methods) commonly used to correct for the number of independent tests were not utilized. In the absence of such a correction, we relied on validation in the independent sample to support the robustness of our findings. Due to the large dependence among tests, the findings reported here likely represent a single underlying phenomenon, and subsequent studies will be necessary to gain a better understanding of these observations.

Finally, the differences between the discovery and validation samples should also be noted. Key characteristics such as age, sex, and alcohol consumption levels differed between the study samples, suggesting that both samples may represent different populations of alcoholics. Moreover, identical measures for negative craving and return to drinking were not available in the validation cohort, limiting our ability to directly replicate the findings in the discovery cohort. Yet, consistent findings acquired in samples differing in those characteristics and with different measured assessments of the same underlying phenotypes can be interpreted as validation, rather than direct replication of our findings; this is true particularly regarding the phenotypes of alcohol dependence and negative craving. Regarding return to drinking, length of sobriety before treatment was chosen as a surrogate measure for time until relapse, under the assumption that *PDYN* variation may be associated with earlier return to drinking at any time. It seemed reasonable to assume that these phenotypes would be correlated, although, in fact, these phenotypes are targeting events (sobriety) before and after treatment, which may potentially be driven by different mechanisms. Therefore the differences in association found between the rs2281285 G allele and length of sobriety in male and female alcoholics before and after treatment may be indicative of a different underlying biology. Further studies are needed to investigate whether these results are due to the fact the phenotypes are distinct, or caused by differences in clinical characteristics between the discovery and validation samples (e.g. differences in levels of alcohol consumption), or simply due to false positive associations. However, despite the differences in study samples and the smaller size of the validation sample, we were able to validate the magnitude and direction of many of the discovered associations regarding the alcohol dependence and negative craving phenotypes. This consistency of results across samples from populations with differing clinical characteristics highlights the robustness of these findings.

In conclusion, our findings support growing evidence that the dynorphin-kappa-opioid system is a critical component linking negative emotions with substance dependence^51^,^52^,^53^. Our findings expend contemporary knowledge by demonstrating that *PDYN* effects on alcohol use disorders differ between males and females, and this pattern of this difference appears to depend on phenotype. Although the exact mechanisms of the reported associations are yet unclear, the results of this study suggest the following directions for future research. First, a search for intermediate phenotypes associated with alcohol dependence subtypes and relevant comorbidities are needed, using meta-analyses and/or combined data sets of subjects with genome wide data to allow increased sample size. Second, *PDYN* sequencing in alcoholics with the proper clinical assessments and treatment outcome data will provide more comprehensive coverage of potentially causal variants and allow testing for association with the above mentioned phenotypes. Last but not least, functional analyses of biological pathways and potential cis and trans regulatory mechanisms involving *PDYN* brain expression are necessary to disentangle the meaning and examine potential causal mechanisms of the differential sex association.

## Additional Information

**How to cite this article**: Winham, S. J. *et al.* Associations of prodynorphin sequence variation with alcohol dependence and related traits are phenotype-specific and sex-dependent. *Sci. Rep.* **5**, 15670; doi: 10.1038/srep15670 (2015).

## Supplementary Material

Supplementary Materials

## Figures and Tables

**Table 1:** Demographic characteristics of the alcohol dependent cases and description of investigated phenotypes.

Variable	Discovery Sample	Replication Sample	P-value
Demographics*			
Age at admission (mean ± SD)	49.1 ± 12.1	43.5 ± 10.4	**<0.0001**
Sex (N and % male)	554 (67.9%)	347 (74.3%)	**0.018**
Average drinks/day at admission (mean ± SD)	8.4 ± 6.4	14.1 ± 8.5	**<0.0001**
**Number of subjects investigated for each phenotype**			
Alcohol dependence (cases/controls)	816/1248	467/431	
Negative craving	196	417	
IDTS negative subscale (mean ± SD)	47.9 ± 21.0	N/M	
SSAGA question: Drinking to avoid emotional and physical symptoms of withdrawal (N and %)	N/M	304 (72.9%)	
Return to drinking	202	409	
Relapse after treatment (N and %)	92 (45.5)	N/M	
Maximum length sobriety in months (mean ± SD)	N/M	22.4 ± 47.9	

^*^Demographic data are presented for alcohol dependent subjects only. Demographic data of controls are presented in previous publications^9^,^10^. N/M, not measured.

**Table 2:** Association of *PDYN* variant rs2281285 with alcohol dependence, negative craving, and time until relapse in the discovery (Mayo) sample, stratified by sex.

Phenotype	Sex	N case/control	MAF (case/control)	Effect Size*	95% CI	P-value
Alcohol Dependence	Both††	816/1248	0.173/0.142	1.30	(1.07, 1.58)	**0.008**
	Male†	554/603	0.171/0.129	1.49	(1.15, 1.93)	**0.002**
	Female†	262/645	0.176/0.154	1.07	(0.79, 1.45)	0.684
Negative Craving	Both††	196	0.157	6.95	(1.55, 12.34)	**0.012**
	Male†	129	0.146	5.14	(-1.34, 11.61)	0.123
	Female†	67	0.179	10.16	(0.44, 19.88)	**0.045**
Time Until Relapse	Both††	202	0.178	1.42	(1.00, 2.00)	**0.048**
	Male†	134	0.157	1.17	(0.73, 1.89)	0.502
	Female†	68	0.221	1.72	(1.03, 2.85)	**0.037**

^*^Type of effect size measure is phenotype-specific: alcohol dependence = odds ratio, negative craving = beta (unstandardized, ITDS 0-100 point scale), time until relapse = hazard ratio. Statistically significant associations (p < 0.05) are presented as bold and underlined; trend for association values (p < 0.1) are underlined.

^†^Analyses were adjusted for age.

^††^Analyses were adjusted for age and sex.

**Table 3:** Association of *PDYN* variant rs2281285 with alcohol dependence, drinking to avoid emotional discomfort, and length of sobriety in the validation (German) samples, stratified by sex.

Phenotype	Sex	N case/control	MAF case/control	Effect size*	95% CI	P-value
Alcohol Dependence	Both††	467/431	0.163/0.148	1.18	(0.91, 1.54)	0.223
	Male†	347/224	0.166/0.129	1.35	(0.96, 1.91)	0.086
	Female†	120/207	0.154/0.169	0.94	(0.61, 1.47)	0.808
Drinking to avoid emotional/physical discomfort	Both††	417	0.173	2.29	(1.08, 4.85)	**0.030**
	Male†	311	0.174	1.79	(0.80, 4.01)	0.158
	Female†	102	0.175	7.11	(0.88, 57.40)	0.066
Longest period of sobriety any time before treatment	Both††	409	0.172	10.064	(1.55, 18.57)	**0.0209**
	Male†	306	0.175	13.487	(5.18, 21.79)	**0.0016**
	Female†	103	0.171	–2.482	(–26.55, 21.59)	0.8403

^*^Effect size measure is dependent on phenotype; alcohol dependence = odds ratio, drinking to avoid emotion discomfort = odds ratio, length of sobriety = beta (months). Statistically significant associations (p < 0.05) are presented as bold and underlined; trend for association values (p < 0.1) are underlined.

^†^Analyses were adjusted for age.

^††^Analyses were adjusted for age and sex.
