# Supplementary material for: Associations of prodynorphin sequence variation with alcohol dependence and related traits are phenotype-specific and sex-dependent
Source: Sci Rep. 2015 Oct 27;5:15670. doi: 10.1038/srep15670 (PMC4621530; doi:10.1038/srep15670)
Supplement: Supplementary Materials [file srep15670-s1.doc]

**Associations of prodynorphin sequence variation with alcohol dependence and related traits are phenotype-specific and sex-dependent.**

Stacey J. Winham^1^, Ulrich W. Preuss^2^, Jennifer R. Geske^1^, Peter Zill^3^, John A. Heit^5^, Georgy Bakalkin^6^, Joanna M. Biernacka^1, 4^, Victor M. Karpyak, MD^4^,*

*^1^Health Sciences Research,* *Mayo Clinic, Rochester, MN, ^2^Department of Psychiatry, Psychotherapy and Psychosomatics, Martin-Luther-University of Halle-Wittenberg, Halle/Saale, Germany,* ^3^LMU Munich, Department of Psychiatry, Section Psychiatric Genetics and Neurochemistry, Munich, Germany*, ^4^Psychiatry and Psychology, Mayo Clinic, Rochester, MN, ^5^Division of Cardiovascular Diseases, Mayo Clinic, Rochester, MN. ^6^Division of Biological Research on Drug Dependence, Department of Pharmaceutical Biosciences, Uppsala University, Uppsala, Sweden.*

**Supplemental Materials**

*Description of the discovery sample study site*

The Department of Psychiatry and Psychology at the Mayo Clinic in Rochester, MN has outpatient treatment programs with and without residing beds directed by Board Certified addiction psychiatrists. The Intensive Addiction Program (IAP) is a 30-day long intensive outpatient treatment program, with residing beds. It serves individuals 18 years and older with chemical dependence and dual-disorders. It includes day time (8:15 a.m.-4:30 p.m.) and evening programming 7 days per week. Program components include Focus Groups, Process Groups, Structured Groups, Experiential Groups, and an Intensive Family Program every other Monday. In-treatment sobriety monitoring includes daily breathalyzer and random Drug Abuse Surveys. Attendance of Alcoholic Anonymous or other support groups is mandatory during treatment.

*Description of the validation sample study site*

In the validation sample, treatment-seeking alcohol-dependent individuals were admitted to the university hospital treatment ward through an outpatient motivational group. Patients underwent medication-assisted alcohol detoxification treatment for a maximum of 7 days. During and after detoxification treatment, the inpatients participate in cognitive-based group therapies, psycho-education on addictive disorders, ergo-, art-, sport and music-therapy. Attendance of self-help and other support groups is mandatory. The complete program lasts between 14 and a maximum of 21 days.

*Assessment of study participants in the discovery sample*

Study assessments were selected from data collected as a part of clinical evaluation and monitoring during treatment in IAP. All patient admitted to IAP are evaluated by board certified addiction psychiatrist to determine presence of alcohol dependence as well as comorbid conditions, including psychiatric conditions and non-alcoholic substance dependence or abuse. In addition, psychometric scales are used for assessment at the time of admission and treatment monitoring. These include but are not limited to the following. Frequency, intensity, and duration of craving were assessed with the Penn Alcohol Craving Scale (PACS) on admission and weekly during treatment. The Inventory of Drug Taking Situations (IDTS) [^3^](#_ENREF_3), [^4^](#_ENREF_4) is used to assess the association of alcohol use with positive or negative emotional states once per treatment episode. The 9 item depression scale from the patient health questionnaire (PHQ-9) [^5^](#_ENREF_5), and the Generalized Anxiety Disorder Assessment (GAD-7) [^6^](#_ENREF_6) are used to assess for the presence and intensity of symptoms of depression and anxiety on admission and prior to discharge.

*Assessment of study participants in the validaiton sample*

In the validation sample, all patients were 18 years or older and met both International Classification of Diseases, Tenth Revision and Diagnostic and Statistical Manual of Mental Disorders, Fourth Edition (DSM-IV) criteria for alcohol dependence; criteria were assessed in a structured interview (Structured Clinical Interview for DSM-IV, German version^7^). Additional characteristics of alcohol dependence, such as withdrawal symptoms, craving and development of tolerance, psychiatric and somatic comorbidity were obtained with the Semi-Structured Assessment for the Genetics of Alcoholism (SSAGA^8^)). A comprehensive psychiatric examination was performed by at least one of the authors (UWP). All patients were examined 2 weeks after admission, free of any withdrawal symptoms or psychopharmacological treatment.

**Supplemental Table 1.** Association of *PDYN* variant rs6132153 with alcohol dependence, negative craving, and time until relapse in the discovery (Mayo) sample, stratified by sex.

| **Phenotype** | **Sex** | **N case/control** | **MAF (case/control)** | **Effect Size*** | **95% CI** | **P-value** |
| --- | --- | --- | --- | --- | --- | --- |
| Alcohol Dependence | Both^††^ | 816/1248 | 0.163/0.143 | 1.22 | (1.00, 1.48) | **0.047** |
|  | Male^†^ | 554/603 | 0.158/0.136 | 1.26 | (0.98, 1.62) | 0.073 |
|  | Female^†^ | 262/645 | 0.174/0.150 | 1.15 | (0.85, 1.57) | 0.363 |
| Negative Craving | Both^††^ | 196 | 0.145 | 5.49 | (-0.13, 11.10) | 0.057 |
|  | Male^†^ | 129 | 0.131 | 2.16 | (-4.64, 8.97) | 0.534 |
|  | Female^†^ | 67 | 0.171 | 10.83 | (0.95, 20.71) | **0.036** |
| Time to Relapse | Both^††^ | 202 | 0.161 | 1.31 | (0.92, 1.88) | 0.131 |
|  | Male^†^ | 134 | 0.142 | 1.08 | (0.66, 1.78) | 0.758 |
|  | Female^†^ | 68 | 0.199 | 1.60 | (0.96, 2.67) | 0.072 |

*Type of effect size measure is phenotype-specific: alcohol dependence=odds ratio, negative craving=beta (unstandardized, ITDS 0-100 point scale), time until relapse=hazard ratio. Statistically significant associations (p<0.05) are presented as bold and underlined; trend for association values (p<0.1) are underlined.

^†^Analyses were adjusted for age

^††^Analyses were adjusted for age and sex

**Supplemental Table-2** Association of the *PDYN* gene-spanning haplotype (rs6045868-rs2235751-rs2281285) with alcohol dependence in the discovery cohort. P-values for the global haplotype test (4 df) are presented for the full sample, males, and females in the column headings. P-values within the table are specific to each haplotype.

|  |  |  | Full sample (P=7.9E-4)* | | | Males (P=2.0E-4)^†^ | | | Females (P=0.70)^†^ | | |
| --- | --- | --- | --- | --- | --- | --- | --- | --- | --- | --- | --- |
| rs6045868 (G/A) | rs2235751 (A/G) | rs2281285 (A/G) | Frequency | Score | P-value | Frequency | Score | P-value | Frequency | Score | P-value |
| G | A | A | 0.64 | -1.74 | **0.08** | 0.65 | -2.13 | **0.03** | 0.64 | -0.11 | 0.91 |
| A | A | A | 0.10 | -0.81 | 0.42 | 0.10 | -0.35 | 0.73 | 0.11 | -0.95 | 0.34 |
| G | G | A | 0.09 | 0.34 | 0.73 | 0.09 | -0.39 | 0.69 | 0.09 | 0.86 | 0.40 |
| A | G | G | 0.14 | 1.23 | 0.21 | 0.14 | 1.64 | 0.10 | 0.14 | -0.04 | 0.97 |

*Analyses were adjusted for age and sex

^†^Analyses were adjusted for age

**References**

1. Flannery BA, Volpicelli JR, Pettinati HM. Psychometric properties of the Penn Alcohol Craving Scale. *Alcohol Clin Exp Res* 1999; **23**(8)**:** 1289-1295.

2. Vander Weg MW, DeBon M, Sherrill-Mittleman D, Klesges RC, Relyea GE. Binge drinking, drinking and driving, and riding with a driver who had been drinking heavily among Air National Guard and Air Force Reserve Personnel. *Mil Med* 2006; **171**(2)**:** 177-183.

3. Annis HM, Martin, G. . Inventory of Drug-Taking Situations. *Addiction Research Foundation*: Toronto, Canada, 1985.

4. Annis HM, Turner NE, Sklar SM. Inventory of Drug-Taking Situations: User’s Guide. *Addiction Research Foundation, Centre for Addiction and Mental Health*: Toronto, Canada, 1997.

5. Kroenke K, Spitzer RL, Williams JB. The PHQ-9: validity of a brief depression severity measure. *J Gen Intern Med* 2001; **16**(9)**:** 606-613.

6. Spitzer RL, Kroenke K, Williams JB, Lowe B. A brief measure for assessing generalized anxiety disorder: the GAD-7. *Arch Intern Med* 2006; **166**(10)**:** 1092-1097.

7. Wittchen HU, Zaudig M, Fydrick T. SKID-I/II: Strukturiertes klinisches Interview für DSM-IV. Göttingen: Hogrefe. 1996.

8. Bucholz KK, Cadoret R, Cloninger CR, Dinwiddie SH, Hesselbrock VM, Nurnberger JI, Jr, Reich T, Schmidt I, Schuckit MA (1994) A new, semi-structured psychiatric interview for use in genetic linkage studies: a report on the reliability of the SSAGA. J Stud Alcohol 1994; **55:**149–158.
